# Impact of combined oral contraceptives and progestin‐only pills on psychological and sexual well‐being of women with endometriosis: A systematic review

**DOI:** 10.1111/aogs.70183

**Published:** 2026-03-12

**Authors:** Noemi Salmeri, Martina Piccini, Francesca Caprara, Edgardo Somigliana, Paola Viganò, Paolo Vercellini

**Affiliations:** ^1^ Department of Clinical Sciences and Community Health Università degli Studi Milan Italy; ^2^ Department of Maternal and Child Health Fondazione IRCCS Ca' Granda Ospedale Maggiore Policlinico Milan Italy

**Keywords:** contraceptives, endometriosis, pain, psychology, systematic review, tolerability

## Abstract

**Introduction:**

Women with endometriosis frequently experience psychological and sexual concerns. Combined oral contraceptives (COCs) and progestin‐only pills (POPs) represent first‐line treatments for the disease, yet their potential negative impact on mood and sexuality remains debated.

**Material and Methods:**

We conducted a systematic review of PubMed, Embase, and Scopus up to August 1st, 2025, to assess whether COCs or POPs adversely affect psychological well‐being or sexual function in this population. Eligible studies included randomized controlled trials (RCTs) and non‐randomized studies comparing COCs or POPs with no treatment in women with endometriosis. Outcomes of interest were adverse event rates and patient‐reported measures of mood and sexual health. Risk of bias was assessed with RoB 2 and ROBINS‐I. Given heterogeneity in outcome measures, we applied vote counting by direction of effect and harvest plots, with confidence intervals (CIs) calculated using the Wilson method, in line with Cochrane guidance. The study protocol was prospectively registered on PROSPERO (CRD420250656420).

**Results:**

Of 1424 records screened, seven studies (four RCTs, three observational) met the inclusion criteria. Risk of bias was low in RCTs and moderate‐to‐serious in observational studies. Six of seven (pooled proportion: 86%; 95% CI: 49%–97%) reported no increased risk of psychological dysfunction in users. None reported increased sexual dysfunction (100%; 95% CI: 65%–100%). Most studies reported significant pain reduction with treatment. Six studies specifically reported on dyspareunia, all of which showed a benefit of treatment over placebo (100%; 95% CI: 61%–100%). No discontinuations for psychological or sexual adverse events were observed when pain remission was achieved.

**Discussion:**

Despite the small number of studies, variability in measurement tools, and short follow‐up (mean 6 months), the evidence suggests that COCs and POPs are generally well tolerated regarding psychological and sexual health, supporting their continued use for long‐term management provided pain remission is achieved.

**Conclusions:**

In women with endometriosis, current evidence does not support a consistent increase in negative psychological or sexual outcomes among COCs or POPs users compared with non‐users.

## Graphical abstract

Hormonal treatments for endometriosis (combined oral contraceptives/progestin‐only pills) do not show increased psychological or sexual risks compared with placebo. When pain is effectively controlled, these therapies appear well tolerated and may improve overall well‐being.

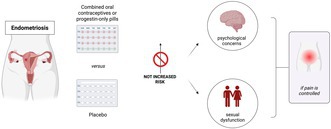

**Abbreviations**
AEs: adverse events
COCs: combined oral contraceptives
CPP: chronic pelvic pain
FSDS: Female Sexual Distress Scale
FSFI: Female Sexual Function Index
GnRH: gonadotropin‐releasing hormone
NSAIDs: non‐steroidal anti‐inflammatory drugs
OR: odds ratio
POPs: progestin‐only pills
PROMs: patient‐reported outcome measures
QoL: Quality of Life
RCT: randomized controlled trial
SF‐36: Short Form‐36 Health Survey
VAS: visual analogue scale
WHOQOL‐BREF: World Health Organization Quality of Life Instrument, Short Form

**Key message**

Concerns persist that combined oral contraceptives or progestin‐only pills may negatively affect psychological well‐being or sexual function in endometriosis. Current evidence indicates these agents do not meaningfully worsen psychological or sexual outcomes and are generally well tolerated when pain improves.

## 1 INTRODUCTION

Hormonal contraception was introduced in the early 20th century and represented a milestone in both reproductive health and women's rights.^1^, ^2^ Over time, combined oral contraceptives (COCs) and progestin‐only pills (POPs) have evolved from contraceptive methods to therapeutic agents for several gynecological conditions.^3^ Among these, endometriosis has become a key indication, with international guidelines recommending COCs or POPs as first‐line therapy.^4^, ^5^ By suppressing ovulation, preventing retrograde menstruation, and reducing cyclic peritoneal inflammation, they can relieve pelvic pain and possibly disrupt disease progression.^6^

Alongside the therapeutic benefits of hormonal contraceptives, concerns have emerged about their tolerability and side effect profiles.^7^ While safety concerns such as thromboembolic risk and cancer have been extensively investigated,^8^, ^9^ evidence on mood and sexual side effects, which are often interrelated, remains limited.

Epidemiological studies have suggested a possible association between hormonal contraceptive use and depression, particularly among adolescents.^10^ In adolescence, these effects may be further amplified by interference with brain maturation and behavior, which are shaped by endogenous hormonal fluctuations during puberty.^11^ Sexual dysfunction in hormonal contraceptive users has been primarily attributed to reduced androgen availability, particularly decreased free testosterone resulting from inhibition of ovarian and adrenal androgen synthesis and increased sex hormone‐binding globulin levels.^12^, ^13^

However, the overall evidence on the impact of oral contraceptives on mental health and sexual function remains inconclusive, with inconsistent findings across different studies.^11^, ^14^

In women with endometriosis, the interpretation of treatment tolerability is particularly challenging. The disease itself is inherently associated with emotional distress and sexual dysfunction,^15^ making it difficult to disentangle disease‐related symptoms from potential effects of hormonal contraceptives.^16^ The side effects profile of COCs and POPs is an utmost relevant aspect in endometriosis management, given the necessity to ensure a long‐lasting adherence for adequate control of the disease.

To address this uncertainty and provide evidence to guide clinical management, we conducted a systematic review restricted to studies in patients with endometriosis, comparing psychological and sexual outcomes between users and non‐users of COCs or POPs. Our aim was to provide evidence‐based insights into the risk–benefit profile of these first‐line treatments in a population already at high risk for psychosocial and sexual health concerns.

## 2 MATERIAL AND METHODS

This systematic review was conducted in accordance with the Preferred Reporting Items for Systematic Reviews and Meta‐Analyses (PRISMA) and the Meta‐analysis Of Observational Studies in Epidemiology (MOOSE) guidelines.^17^, ^18^ The study protocol was prospectively registered on PROSPERO (CRD420250656420).

Two reviewers (F.C. and M.P.) systematically searched PubMed, Embase, and Scopus up to August 1st, 2025. Discrepancies were resolved by a third reviewer (N.S.). The search strategy combined Medical Subject Headings (MeSH) and equivalent free‐text keywords (Table S1).

Eligibility was defined using the PICOS framework.^19^ The population included reproductive‐age women with a diagnosis of endometriosis, without restrictions on disease location or stage. Interventions consisted of combined oral contraceptives (COCs) or progestin‐only pills (POPs), with no limits on the type of progestin or estrogen component. Studies evaluating other forms of hormonal contraception, including gonadotropin‐releasing hormone (GnRH) analog, subdermal implants, hormonal intrauterine devices, injectables, or vaginal rings, or studies in which COCs or POPs were used as add‐back therapy during GnRH analogue treatment, were excluded.

Comparators included no treatment or placebo; concomitant on‐demand use of non‐steroidal anti‐inflammatory drugs (NSAIDs) was permitted and not considered an exclusion criterion. Any study using another hormonal therapy as a comparator for the management of endometriosis‐associated symptoms was excluded. Eligible outcomes encompassed psychological measures, including assessments of mood disorders (e.g. depression, anxiety), psychological distress, emotional well‐being, and mental domains of quality of life, reported through validated patient‐reported outcome measures (PROMs) or documented as psychological adverse events (e.g. mood swings, irritability, depressive symptoms). Sexual outcomes included measures of sexual function, satisfaction, desire, arousal, orgasm, dyspareunia, or overall sexual well‐being, evaluated in validated PROMs or reported as adverse events classified as sexual dysfunction.

Eligible study designs comprised randomized controlled trials and non‐randomized interventional studies, including prospective or retrospective observational designs. Descriptive studies (case reports, case series) and non‐original research (reviews, abstracts, editorials, commentaries) were excluded. All included studies were required to be published in peer‐reviewed journals with full‐text available in English.

All records were screened independently by two reviewers (F.C. and M.P.), first by title and abstract, followed by full‐text assessment against the eligibility criteria. Any disagreements were resolved through discussion with a third reviewer (N.S.).

The following information was extracted and tabulated for each included study: (i) first author and year of publication; (ii) study design, country, and study period; (iii) total sample size and number of participants in each study arm (intervention vs. comparator); (iv) diagnostic method used for endometriosis; (v) study inclusion and exclusion criteria; (vi) treatment characteristics, including the type of estrogen–progestin combination in COCs and the type of progestin in POPs, as well as treatment duration; (vii) methods used to assess the primary outcomes including the type of PROMs (with total and subscale scores), crude proportions and percentages of adverse events (AEs), and rates of treatment discontinuation due to AEs, along with p‐values for between‐group comparisons; (viii) methods adopted to evaluate intervention efficacy in the treatment group were also collected, including changes in endometriosis‐associated pain assessed by visual analogue scale (VAS), numeric rating scale, or other validated PROMs; (ix) bleeding patterns and achievement of amenorrhea during treatment; (x) other potential confounding factors.

### 2.1 Risk of bias and trustworthiness assessment

Risk of bias was independently assessed by two reviewers (F.C. and M.P.), with any discrepancies resolved by a third reviewer (N.S.).

RCTs were evaluated using the Risk of Bias 2 (RoB 2) tool^20^ and further assessed for methodological trustworthiness according to the criteria proposed by the Obstetrics and Gynecology Editors' Integrity Group.^21^

Observational studies were assessed using the Risk Of Bias In Non‐randomized Studies—of Interventions (ROBINS‐I) tool.^22^

### 2.2 Data analysis

Following the methodological recommendations of the Cochrane Handbook for Systematic Reviews of Interventions, Chapter 12,^23^ when studies retrieved according to pre‐specified PICOS criteria report heterogeneous effect measures or provide incomplete outcome or effect estimates, a meta‐analysis should not be performed, and alternative synthesis methods should be applied. In accordance with these recommendations and given that the included studies either reported only the direction of effect or displayed inconsistency in effect measures and outcome reporting, we applied vote counting based on the direction of effect as the primary synthesis method.

Each effect estimate was classified as showing either an improvement or a worsening of psychological or sexual function in women with endometriosis treated with COCs or POPs, compared with those not receiving these treatments. This approach enabled the creation of a standardized binary metric based solely on effect direction, independent of effect size or statistical significance. When no effect was reported, the study was considered to contribute equally to the null hypothesis of no difference.

The pooled proportion of effects indicating a worsening of psychological or sexual function in the treatment group relative to the comparator was calculated as: *p‐ = u−/n*, where *u‐* represents the number of effects indicating a higher risk of psychological or sexual dysfunction in the treatment group, and *n* is the total number of studies included. Similarly, the pooled proportion of effects indicating improvement was calculated as: *p+ = u+/n*, where *u+* represents the number of effects indicating a lower risk of psychological or sexual dysfunction in the treatment group.

Two‐sided binomial probability tests (sign tests) were used to determine whether the observed proportions differed significantly from the null expectation of random directionality. To ensure a conservative inference framework, the null hypothesis was set as an equal probability of positive and negative findings. Ninety‐five percent confidence intervals (95% CI) for the observed proportions were calculated using the Wilson method.^24^

Sensitivity analyses were conducted by restricting the synthesis to RCTs only, and subgroup analyses were performed based on treatment efficacy. All statistical analyses were performed using StataNow/SE 18.5.^25^

To visually summarize the findings, and in line with the methodology previously described,^26^ harvest plots were created to depict psychological and sexual outcomes in COCs or POPs users vs. non‐users, categorized as negative, null, or positive effects. Plots were created using the ggplot2 package in R version 4.3.1^27^ within the RStudio environment version 2023.06.1 + 524 (Posit PBC, Boston, MA, USA).

All results were reported in accordance with the Synthesis Without Meta‐analysis (SWiM) guideline.^28^

## 3 RESULTS

**FIGURE 1:**
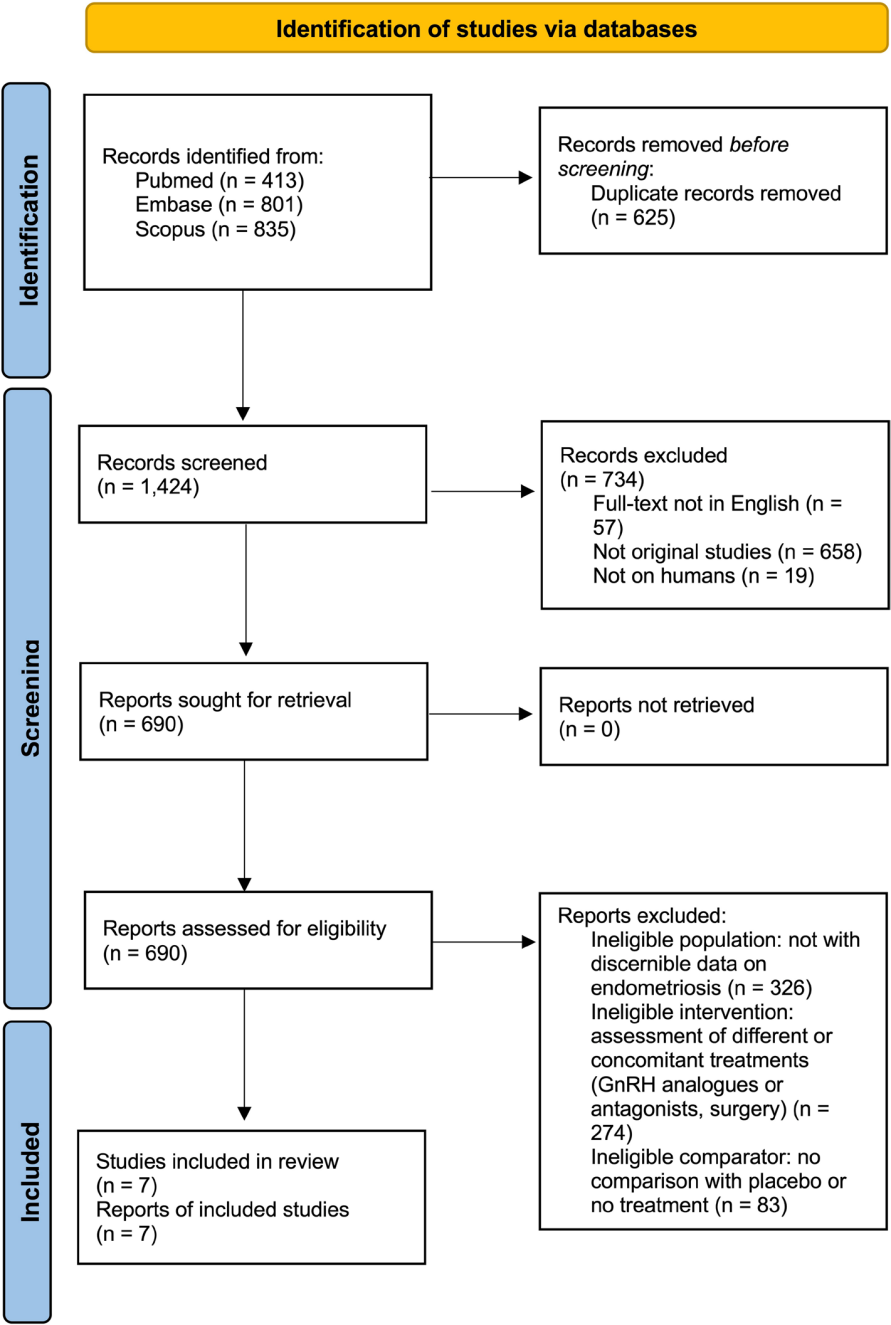
PRISMA 2020 flow diagram.

Out of 2049 records identified, 1424 remained after duplicate removal and were screened. Following title/abstract and full‐text assessment, seven studies met PICOS criteria and were included in the final analysis: four RCTs and three observational studies (two prospective, one retrospective).^29^, ^30^, ^31^, ^32^, ^33^, ^34^, ^35^ The PRISMA 2020 flow diagram is presented in Figure 1.

### 3.1 Studies overview

Study characteristics according to PICOS are summarized in Table 1.

**TABLE 1:** Characteristics of included studies according to PICOS (*n* = 7).

First author (year)	Population		Intervention	Comparator	Outcomes			Study design and setting
	Endometriosis diagnosis	Inclusion and exclusion criteria			Primary: psychological and sexual domains	Secondary: treatment efficacy	Primary outcome in the original study	
Caruso et al. (2015)^30^	Based on clinical symptoms (dysmenorrhea, CPP, deep dyspareunia, and dyschezia) and US confirmation according to ESHRE 2014 guidelines	Inclusion: 18–39 years; endo‐associated CPP; sexually active (> = 1 sexual activity/month) Exclusion: non‐organic sexual dysfunction; partner affected by sexual dysfunction; wash‐out from GnRH analogue <6 months or COCs <3 months	Dienogest 2 mg/daily, continuous regimen for 6 months	No treatment (on‐demand NSAIDs, including only: paracetamol, ibuprofen, ketoprofen, ketorolac, diclofenac by oral route)	Mood: self‐administered SF‐36 (subscales on mental aspects: vitality, social activity, emotional role, mental health) Sexual function: self‐administered FSFI (total scores and 6 sub‐scales: desire, arousal, lubrication, orgasm, satisfaction, pain); self‐administered FSDS (total score)	Efficacy on chronic pain: changes from baseline in VAS for pelvic pain; % reduction in pelvic pain, dysmenorrhea, dyspareunia; amenorrhea achievement (*n*, %)	QoL and sexual function in intervention vs. comparator	Design: Prospective observational study Country: Italy Time: NR
Caruso et al. (2020)^32^	Based on clinical symptoms (dysmenorrhea, CPP, deep dyspareunia, and dyschezia) and US confirmation according to ESHRE 2014 guidelines	Inclusion: 18–37 years; endo‑associated CPP refractory to on‐demand NSAIDs in the last 15 months to 8 years; sexually active (≥1 sexual activity/month) Exclusion: non‐organic sexual dysfunction; partner affected by sexual dysfunction; wash‐out from GnRH analogue <6 months or COCs <3 months	COCs containing 1.5 mg estradiol and 2.5 mg NOMAC, 24 + 4 regimen for 6 months	No treatment (on‐demand NSAIDs, including only: paracetamol, ibuprofen, ketoprofen, ketorolac, diclofenac by oral route)	Mood: self‐administered SF‐36 (total score for mental subscales) Sexual function: self‐administered FSFI (total score); self‐administered FSDS (total score); diary on the monthly frequency of sexual activity	Efficacy on chronic pain: changes from baseline in VAS for pelvic pain; % reduction in pelvic pain, dysmenorrhea, dyspareunia	QoL and sexual function in intervention vs. comparator	Design: Prospective observational study Country: Italy Time: October 2017 to March 2019
Cevik and Taylor (2025)^35^	Based on ICD‐10th codes (code N80)	Exclusion: <18 or >45 years; malignancy; use of psychotropic medications other than antidepressants; diagnosis of a psychiatric pathology other than depressive disorders; adenomyosis only	Any COCs, for any duration during the retrospective study period (>50% first generation)	Non‐COCs users (unclear definition of concurrent treatments during the retrospective study period)	Mood/sexual function: *n* (%) with a diagnosis of depressive disorders based on ICD‐10th codes (code F32.0–F32.9); *n* (%) of treatment discontinuation due to mood lability/depression and to decreased libido	NR	Role of mood lability in treatment‐users and discontinuation	Design: Retrospective cohort study Country: United States Time: institutional register data from 2012 through 2024
Harada et al. (2024)^34^	Based on surgery or imaging (for endometrioma) or gynecological examination	Inclusion: ≥20 years; regular menstrual cycles (25–38 days); VAS >4 for CPP; BMI <30 kg/m^2^ Exclusion (main): AUB <6 months before screening; endometrioma >10 cm or with solid components; clinically diagnosed with severe depression ≤1 year before screening or currently; wash‐out from: any surgery <2 months; HT or anxiolytics <1 month; GnRH analogs <2 months	COCs containing 15 mg estetrol and 3 mg DRSP, 24 + 4 regimen for 6 cycles	Oral placebo tablets daily, 28 for 6 cycles	Mood and sexual function: AEs (*n*, %) for psychological disorders (including libido decreased)	Efficacy on multiple pain domains: changes from baseline in VAS; *n* (%) achieving 30% and 50% reduction in pelvic pain; gynecological examination for changes in cul‐de‐sac induration, pelvic tenderness, and uterine mobility	Efficacy and safety of treatment vs. placebo Sample size calculation based on the efficacy outcome (reduction of VAS >2)	Design: Randomized, double‐blind, placebo‐controlled, clinical trial Country: Japan (25‐centers) Time: August 2nd 2021 to November 29th 2022
Lang et al. (2018)^31^	Based on laparoscopy or laparotomy within 10 years before study entry	Inclusion: 18–45 years; VAS ≥3 for CPP Exclusion: AUB; amenorrhea >3 months; wash‐out from GnRH agonists <6 months, long‐acting agents <3 months, short‐acting agents <1 month	Dienogest 2 mg/daily, continuous regimen for 6 months	Oral placebo tablets daily for 6 months (identical appearance to treatment)	Mood: self‐administered SF‐36 (total score for mental subscales)	Efficacy on pelvic pain: changes from baseline in VAS; *n* (%) achieving 25% and 75% reduction in pelvic pain; changes in B&B severity profile scores; intake of analgesics (ibuprofen 200 mg tablets)	Efficacy and safety of treatment vs. placebo Sample size calculation based on the efficacy outcome (reduction of VAS >1)	Design: Randomized, double‐blind, placebo‐controlled trial Country: China (23 centers) Time: March 2013 to April 2015
Mehdizadeh Kashi et al. (2022)^33^	Confirmed by laparoscopic surgery performed prior to enrollment to restore normal anatomy of the reproductive organs	Inclusion: 18–45 years; presence of dysmenorrhea, dyspareunia, dysuria, dyschezia, pelvic pain; BMI 18.5–29.9 kg/m^2^ Exclusion: pelvic pain originating from other sources; any other gynecological condition (benign or malignant); wash‐out from GnRH analogue or other HT <3 months	Dienogest 2 mg/daily, continuous regimen for 6 months	Oral placebo tablets daily for 6 months	Mood: persian version of the WHOQOL‐ BREF questionnaire (6 items assessing psychological health)	Efficacy on pelvic pain: changes from baseline in VAS	Efficacy of treatments vs. placebo No sample size calculation	Design: Randomized, double‐blind, placebo‐controlled trial; 3‐arms Country: Iran Time: March 30th 2018 to March 21st 2020
			COCs containing 0.03 mg Ethinyl Estradiol and 0.3 mg LNG, mg/daily, continuous regimen for 6 months					
Strowitzki et al. (2010)^29^	Based on diagnostic laparoscopy within 12 months before study entry	Inclusion: 18–45 years; VAS ≥3 for CPP Exclusion: amenorrhea ≥3 months; wash‐out from HT <1–6 months (dependent on the class of agent); any gynecological comorbidity at examination	Dienogest 2 mg/daily, continuous regimen for 3 months, after a 4‐week treatment‐free period	Oral placebo tablets daily for 3 months	Mood: changes in self‐administered SF‐36 (individual score for role emotional); *n* (%) with depression (as adverse event)	Efficacy on pelvic pain: changes from baseline in VAS; intake of analgesics; B&B severity profile scores	Efficacy and safety of treatment vs. placebo Sample size calculation based on the efficacy outcome (reduction of VAS and intake of analgesics)	Design: Randomized, double‐blind, placebo‐controlled trial Country: China (23 centers) Time: March 2013 to April 2015

Abbreviations: AEs, adverse events; B&B, Biberoglu and Behrman scale; CPP, chronic pelvic pain; COCs, combined hormonal contraceptives; DRSP, Drospirenone; E4, Estetrol; EE, Ethinylestradiol; FSDS, Female Sexual Distress Scale; FSFI, Female Sexual Function Index; FU, follow‐up; GnRH, Gonadotropin‐Releasing Hormone; ICD‐10th, International Classification of Diseases, 10th Revision; LNG, Levonorgestrel; NSAIDs, Nonsteroidal Anti‐Inflammatory Drugs; NOMAC, Nomegestrol Acetate; QoL, Quality of Life; SF‐36, Short Form‐36 Health Survey; US, ultrasound; VAS, Visual Analogue Scale; WHOQOL‐BREF, World Health Organization Quality of Life Instrument, Short Form.

#### 3.1.1 Population

Endometriosis diagnosis criteria were fairly homogeneous. Three RCTs required surgical confirmation^29^, ^31^, ^33^ while the remaining RCT^34^ and the two prospective studies^30^, ^32^ accepted imaging or clinical criteria (i.e., endometriosis‐associated pain symptoms). The retrospective study^35^ adopted ICD‐10 code for endometriosis (N80), with no restrictive inclusion criteria. Conversely, all RCTs^29^, ^31^, ^33^ applied well‐defined eligibility criteria, specifically moderate‐to‐severe chronic pelvic pain (CPP) (VAS ≥30 mm over 100 mm or numeric rating scale ≥3/10) and a wash‐out period from prior hormonal therapy (1–3 months for COCs/POPs, 2–6 months for depot formulations/GnRH analog). One RCT also excluded women with diagnosis of severe depression within the past year.^34^

#### 3.1.2 Intervention and comparator

The two prospective studies^30^, ^32^ compared either cyclic COCs containing natural estradiol and nomegestrol acetate or continuous dienogest vs. no treatment (with NSAIDs allowed) for 6 cycles. The retrospective study^35^ compared COCs users (mostly first‐generation, unknown regimen) vs. non‐users over a 12‐year register, with surgical treatments allowed during the study period and more frequently reported in COCs users. Among RCTs, one tested cyclic COCs with estetrol/drospirenone for six cycles,^34^ two tested continuous dienogest for 3–6 months^29^, ^31^ and one included three arms (continuous dienogest or ethinylestradiol + levonorgestrel, and placebo) for six cycles.^33^

#### 3.1.3 Outcome(s)

There was substantial heterogeneity in the measures used to assess psychological and sexual outcomes. The two prospective studies^30^, ^32^ employed standardized PROMs, specifically the psychological subscales of the Short Form‐36 Health Survey (SF‐36), as well as the Female Sexual Function Index (FSFI) and the Female Sexual Distress Scale (FSDS) for sexual function. The retrospective study assessed mood disorders using ICD‐10 codes and reported on treatment discontinuation due to mood lability and on reduced libido.^35^ The RCTs reported between‐group differences in psychological AEs and/or PROMs scores using either the SF‐36 or the Persian version of the World Health Organization Quality of Life Instrument, Short Form (WHOQOL‐BREF).^29^, ^31^, ^33^, ^34^ All studies, except the retrospective one,^35^ reported on dysmenorrhea and other pain‐related outcomes, consistently demonstrating symptom improvement with treatment. However, only one study reported amenorrhea as being achieved in most treated participants.^30^ One study did not provide direct measures of efficacy but documented a treatment discontinuation rate exceeding 50% due to inadequate symptom relief.^35^

### 3.2 Risk of bias and trustworthiness

Risk of bias assessments are shown in Figure S1. Trustworthiness assessment for RCTs is reported in Table S2.

Among observational studies, two were rated as having a moderate risk of bias due to selective reporting (Domain 7),^30^, ^32^ while one was rated at serious risk,^35^ with moderate bias in confounding (Domain 1) and exposure classification (Domain 2), and serious bias in deviations from intended interventions (Domain 4) per ROBINS‐I (Figure S1a). Of the four RCTs, one was judged to have low risk of bias,^29^ while the others were at moderate risk due to specific issues: Domain 3 (reporting relevant outcomes only as cumulative AEs^34^), Domain 5 (not reporting between‐group comparisons for all PROMs^31^), and Domains 3 and 4 (per‐protocol analysis only^33^) (Figure S1b).

### 3.3 Summary of results

#### 3.3.1 Psychological outcomes

Psychological outcomes were assessed in all included studies (Table S3). Overall, six of seven studies^29^, ^30^, ^31^, ^32^, ^33^, ^34^ found no increased risk of psychological dysfunction in COCs/POPs users vs. non‐users (pooled proportion: 86%; 95% CI: 49%–97%), and only one^35^ reported an increased risk (14%; 95% CI: 3%–51%; two‐sided binomial test *p* = 0.13).

Three studies assessed psychological dysfunction as an AE. Two RCTs reported similar rates of depression or mood disorders between treatment and placebo groups.^29^, ^34^ In contrast, the retrospective study found a significantly higher risk of mood lability and depression in COCs users vs. non‐users (RR = 1.79; 95% CI: 1.53–2.09).^35^ The proportion of studies reporting increased psychological AEs in the treatment group was therefore 33% (95% CI: 6%–79%; *p* = 1.0). Importantly, both RCTs reporting no AE difference also documented significant pain relief with treatment,^29^, ^34^ whereas the retrospective study reported inadequate symptom relief in 52% of participants, leading to discontinuation.^35^

Five studies evaluated psychological outcomes with PROMs, yielding consistent results.^29^, ^30^, ^31^, ^32^, ^33^ One found significantly lower SF‐36 scores in mental health‐related subscales (social functioning, emotional role, mental health, vitality) in treatment vs. control groups.^30^ Among these five studies adopting PROMs, four (80%; 95% CI: 38%–96%; *p* = 0.38) reported improvement in mental health scores,^30^, ^31^, ^32^, ^33^ and all five (100%; 95% CI: 57%–100%; *p* = 0.06) reported improvement in at least one emotional health subscale.^29^, ^30^, ^31^, ^32^, ^33^

All these studies also reported pain improvement with treatment, with one^30^ documenting amenorrhea achievement in ~80% of participants.

Only the retrospective study reported discontinuation due to psychological side effects (mood lability, depression) in one‐third of users, corresponding to 14% of all studies (95% CI: 3%–51%).^35^ The remaining 86% (95% CI: 49%–97%) did not report such reason for discontinuation (*p* = 0.13). Restricting analysis to the six studies showing significant pain improvement, none reported discontinuation due to psychological side effects (0%; 95% CI: 0%–40%; *p* = 0.03).^29^, ^30^, ^31^, ^32^, ^33^, ^34^

**FIGURE 2:**
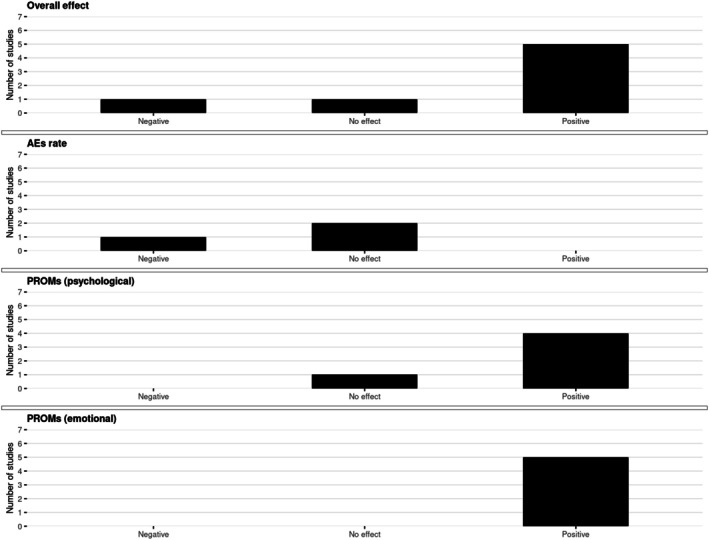
Harvest plots on psychological outcomes. The panel shows harvest plots summarizing the direction of study‐level effects for combined oral contraceptives (COCs) and progestin‐only pills (POPs) on psychological outcomes in women with endometriosis, comparing users with non‐users. Outcomes are grouped as overall effect (*n* = 7), adverse events (AEs) rate (*n* = 3), and patient‐reported outcome measures (PROMs) subdivided into psychological (*n* = 5) and emotional (*n* = 5) domains. For each outcome, the number of studies reporting negative, no effect, or positive effects is depicted.

The harvest plots for psychological outcomes are shown in Figure 2.

#### 3.3.2 Sexual outcomes

All studies reported sexual health‐related data (Table S3). Because dyspareunia encompasses both pain severity and sexual function, changes in this outcome should be interpreted in the context of overall pain control. None found an increased risk of sexual dysfunction in the treatment group (proportion: 100%; 95% CI: 65%–100%; *p* = 0.01). Two studies reported sexual AEs with no significant difference between groups (0%; 95% CI: 0%–66%; *p* = 0.5).^34^, ^35^

Dyspareunia outcomes were reported in all but one study.^35^ All six of these reported a reduction in dyspareunia with treatment (100%; 95% CI: 61%–100%; *p* = 0.03), and five reported a statistically significant difference vs. controls (83%; 95% CI: 44%–97%; *p* = 0.22).^29^, ^30^, ^31^, ^32^, ^33^

The two prospective observational studies used validated PROMs, showing significant improvement in total FSFI and FSDS scores in treated women vs. controls (100%; 95% CI: 34%–100%; p = 0.5).^30^, ^32^ Caruso et al. also found significant improvements in individual FSFI subscales (desire, arousal, lubrication, orgasm, satisfaction, and dyspareunia).

Only one study reported discontinuation due to sexual dysfunction, occurring in 0.3% of COCs users.^35^

**FIGURE 3:**
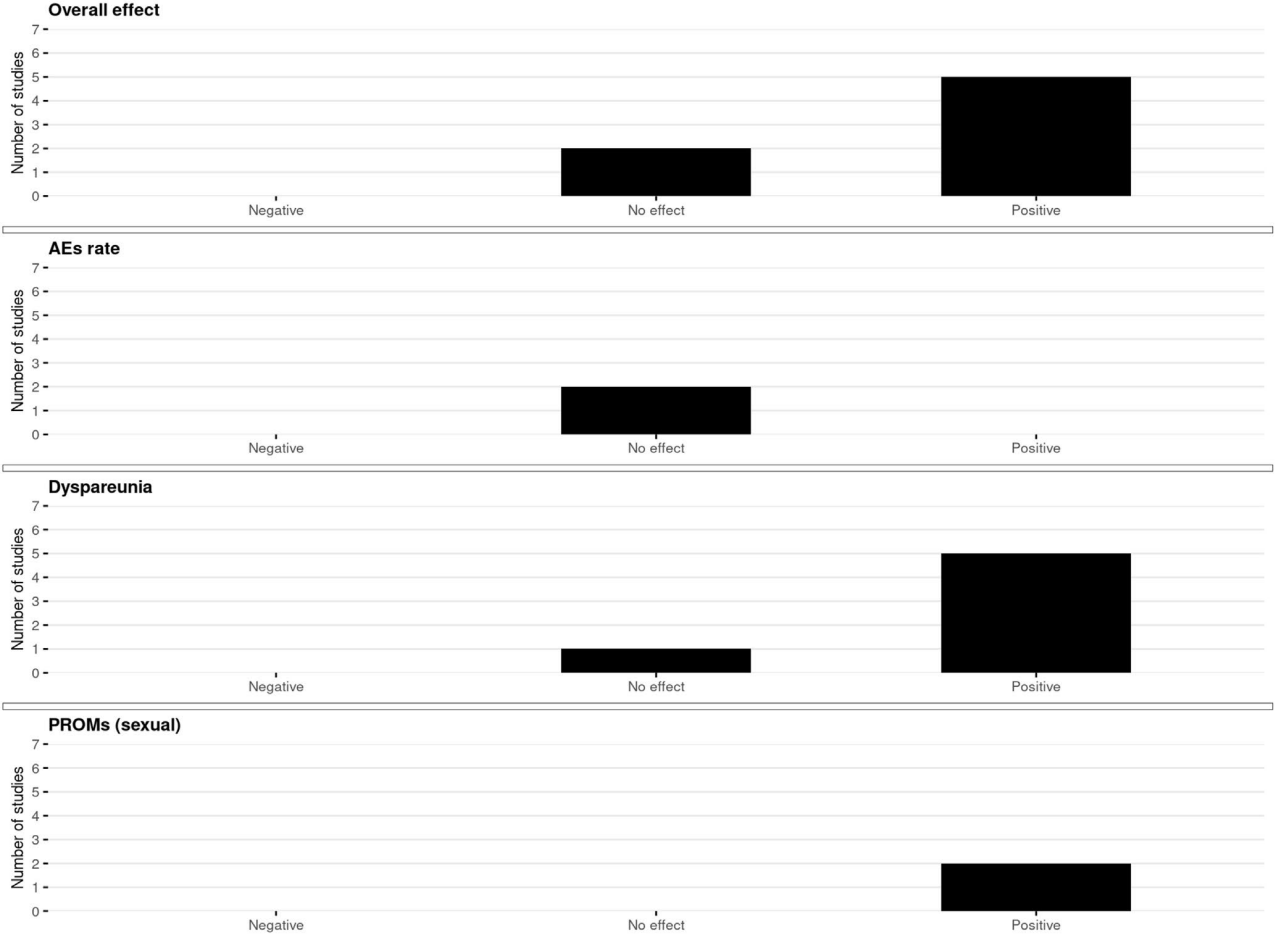
Harvest plots on sexual outcomes. The panel shows harvest plots summarizing the direction of study‐level effects for combined oral contraceptives (COCs) and progestin‐only pills (POPs) on sexual outcomes in women with endometriosis, comparing users with non‐users. Outcomes are grouped as overall effect (*n* = 7), adverse event (AEs) rate (*n* = 2), dyspareunia (*n* = 6), and patient‐reported outcome measures (PROMs) (*n* = 2). For each outcome, the number of studies reporting negative, no effect, or positive effects is depicted.

The harvest plots for sexual outcomes are presented in Figure 3.

## 4 DISCUSSION

In this systematic review, COCs or POPs use in women with endometriosis was not associated with an increased risk of psychological dysfunction compared with non‐use and was associated with a significantly lower likelihood of sexual dysfunction. Most studies reported significant improvements in pain, including dyspareunia, with treatment. None of the included studies that reported adequate pain relief in the treatment group reported treatment discontinuation due to psychological or sexual adverse effects. PROM‐based assessments showed consistent improvement in multiple domains of mental health and sexual function among users compared with non‐users.

The potential side effects of COCs and POPs on mood and sexual function remain a major concern for both clinicians and patients, particularly in the context of endometriosis management. Women with the disease often experience significant social and psychological distress across multiple domains, including mental health and emotional well‐being.^36^ Sexual function is also commonly impaired, with repercussions for partners and relationship quality.^37^

In our review, which included only women with endometriosis, the direction‐of‐effect analysis using a two‐sided binomial test did not indicate an increased risk of mood or sexual dysfunction in COCs or POPs users compared with non‐users. On the contrary, PROMs often showed improvements in psychological and sexual health among users, particularly when treatment achieved clinically meaningful reductions in CPP, a key indicator of therapeutic efficacy.^38^

These findings are consistent with the well‐established interplay between pain, mood, and sexual health. Depression, sexual dysfunction, and pain frequently co‐occur in women. A large systematic review of more than 100 studies reported significant associations between depression and endometriosis‐related pain, including dysmenorrhea, dyspareunia, and non‐cyclical pelvic pain.^39^ In line with this, a path analysis in women with endometriosis identified pelvic pain and dyspareunia as major risk factors for sexual dysfunction, together with anxiety, depression, and poor sleep quality; importantly, greater pain burden correlated with poorer psychological outcomes, whereas effective pain management was linked to measurable mental health benefits.^40^

Indeed, chronic, moderate‐to‐severe, or treatment‐refractory pain is strongly associated with worse depression outcomes, including reduced QoL, impaired work function, and greater healthcare utilization.^41^ These clinical observations are supported by biological evidence that pain and depression share common genetic factors, molecular pathways, and neurotransmitter systems, raising the “chicken‐and‐egg” question as to which comes first.^42^, ^43^ Consistently, in endometriosis, chronic pain‐related phenotypes have been strongly associated with depression in multivariate analyses (OR = 3.61), supported by robust genetic correlations.^44^ Collectively, this evidence reinforces the view that mental health dysfunction in endometriosis may be intrinsically linked to the disease itself and, in some cases, could improve with effective pain control.

One important aspect for clinical management, particularly in endometriosis, is that estrogens generally exert neuroprotective effects, whereas progesterone has been consistently associated with mood alterations. Notably, most cases of depression have been linked to the continuous use of potent progestins in the absence of estrogens.^45^ Indeed, after estrogen doses in contraceptives were substantially reduced post‐1969 to mitigate the risk of venous thromboembolism, reports of depression, anxiety, and weight gain increased among young women.^46^ Of note, in a cohort of young women (mean age ~ 19), COCs users reported higher QoL compared with naturally cycling women, whereas POPs use was associated with more self‐reported mental health diagnoses and altered cortisol levels, underscoring the role of contraceptive type in mental health.^47^ Furthermore, the type of estrogen also matters: Newer formulations containing bioidentical estradiol may be better tolerated than older ethinylestradiol‐based pills, with a weaker reported link to mood problems.^48^ Similarly, regarding sexual function, the type and dose of COCs may be crucial, as different progestins affect androgen metabolism in different ways, while low local estrogen levels can reduce lubrication and arousal, impairing sexual satisfaction and overall function.^13^

In our review, we were unable to draw formulation‐specific conclusions in women with endometriosis. Only one study evaluated COCs combining bioidentical estrogens with a progestin of moderate antiandrogenic activity and reported consistent psychological and sexual health benefits in users compared with non‐users.^32^

The main strength of this review is that it is the first study to include exclusively women with endometriosis, comparing standard treatment with COCs or POPs against no hormonal treatment. This design minimized baseline confounding, as women with endometriosis inherently carry a higher risk of psychological and sexual dysfunction and should not be compared with controls without the disease. Ascertainment bias was reduced because most included studies defined endometriosis using specific criteria, either surgical and histological confirmation or, for ovarian and deep endometriosis, imaging by transvaginal ultrasound and/or magnetic resonance.

Another strength lies in the methodological approach: although quantitative synthesis was not feasible due to heterogeneity, we applied strict Cochrane‐based methods to robustly assess the direction of effect. The vote‐counting approach, while unable to estimate effect size or weight studies by sample size, was appropriate to test our hypothesis, namely, to determine whether COCs or POPs use in women with endometriosis could be associated with mood and/or sexual dysfunction, rather than to estimate a specific effect size.

However, this systematic review has several limitations. First, the relatively small number of studies introduces uncertainty around the estimated proportions, although 95% CIs were calculated using the Wilson method, which provides robust coverage even with small sample sizes.^24^ Second, psychological and sexual dysfunction are multidimensional constructs, and reducing them to binary outcomes may have introduced misclassification bias, potentially oversimplifying complex symptom patterns. Third, both benefits and adverse effects may require time to manifest. In our systematic review, follow‐up in the included RCTs showing no increased risk of depression or sexual dysfunction was limited to 6 months, whereas the only long‐term study reporting an increased risk of mood lability in COCs or POPs users was retrospective.^35^ Importantly, the risk of depression appears to be highest within the first 2 years of treatment initiation,^49^ and up to 30% of users discontinue within 6 months due to mood symptoms,^50^ suggesting that negative effects should already be detectable with shorter follow‐up in RCTs. Consistently, pharmacovigilance data from the Food and Drug Administration (FDA) identified 6502 cases of progestogen‐related depression over 20 years, with onset typically within 180 days and mainly associated with levonorgestrel, medroxyprogesterone, and desogestrel.^45^ In women with endometriosis, a pooled analysis of cohort studies with long follow‐up reported depression in only 1.5% of 21090 patients using COCs and 0.7% of 72451 women without endometriosis, corresponding to a high number needed to harm (431; 95% CI, 324–657).^51^

The retrospective design of the only negative study^35^ also raises concerns, as it provided limited control for key confounders such as symptom relief and concurrent interventions, including surgery.^52^ This is particularly relevant in endometriosis, where the disease trajectory involves exposure to multiple confounding factors, including repeated surgeries, treatment interruptions for fertility purposes, and infertility itself, all factors independently associated with psychological dysfunction.^53^ Taken together, these limitations highlight the methodological challenges of observational studies and support the value of RCTs, which, despite short follow‐up, offered consistent evidence of no increased risk of mood or sexual dysfunction in women with endometriosis using COCs or POPs.

## 5 CONCLUSION

Mood and sexual dysfunction are common in women with endometriosis and should be identified early and addressed across the life course. Potential mood side effects should not discourage prescription of hormonal contraceptives, as overall evidence does not indicate an increased risk of depression; in some cases, hormonal treatment may even be beneficial in mood disorders that disproportionately affect women.^54^

Because of the strong interplay between pain, psychological well‐being, and QoL, achieving bleeding suppression and pain control remains central in endometriosis management. This can often be obtained by following simple clinical rules,^55^ allowing most patients to be managed successfully in the long term.^56^ Formulation and administration route influence tolerability: bioidentical estradiol is generally better tolerated, and administration route modulates hormonal activity and metabolism.^57^ If tolerability issues occur despite optimized therapy, a multimodal approach with targeted management of side effects may be beneficial. Psychological interventions such as cognitive behavioral therapy are effective for chronic pain in general,^58^ and endometriosis‐related pain.^59^ Pelvic floor therapy may be integrated in cases of sexual dysfunction due to pelvic floor spasm. If women prefer to discontinue hormonal treatment, alternatives such as neuromodulation, particularly for those with widespread pain and central sensitization,^60^ or surgical management, when indicated, remain alternatives for achieving symptom control and improving QoL.

With that in mind, and in line with women's reproductive mental health principles^16^ long‐term endometriosis care should move beyond one‐size‐fits‐all symptom control toward tailored strategies that ensure pain relief and tolerability, empowering women and sustaining lifelong well‐being.

## AUTHOR CONTRIBUTIONS

**Noemi Salmeri:** Conceptualization, investigation, project administration, data curation, formal analysis, methodology, software, visualization, resources, writing—original draft. **Martina Piccini:** Investigation, data curation, validation, writing—review and editing. **Francesca Caprara:** Investigation, data curation, validation. **Edgardo Somigliana:** Validation, funding acquisition, writing—review and editing. **Paola Viganò:** Validation, funding acquisition, writing—review and editing. **Paolo Vercellini:** Conceptualization, validation, funding acquisition, writing—review and editing, supervision.

## CONFLICT OF INTEREST STATEMENT

All authors report financial support was provided by Italian Ministry of Health—Current Research IRCCS. The Italian Ministry of Health had no role in the study design, collection, analysis, or interpretation of the data, writing the manuscript, or the decision to submit the paper for publication.

N.S. reports a relationship with the World Endometriosis Society (WES) and the Society for Endometriosis and Uterine Disorders (SEUD) that includes the following: travel reimbursement. E.S. reports a relationship with Ferring, Theramex and IBSA that includes: funding grants. E.S. reports a relationship with IBSA, Gedeon‐Richter and Sandoz that includes: speaking and lecture fees. P.Vi. reports a relationship with Journal of Endometriosis and Uterine Disorders that includes the following: board membership. P.Ve. reports a relationship with Wolters Kluwer UpToDate that includes the following: consulting or advisory. If there are other authors, they declare that they have no known competing financial interests or personal relationships that could have appeared to influence the work reported in this paper.

## Supporting information


Data S1.

## Data Availability

Data will be made available to the editors for review or query upon request.
