# Supplementary material for: Impact of combined oral contraceptives and progestin‐only pills on psychological and sexual well‐being of women with endometriosis: A systematic review
Source: Acta Obstet Gynecol Scand. 2026 Mar 12;105(5):805–18. doi: 10.1111/aogs.70183 (PMC13125366; doi:10.1111/aogs.70183)
Supplement: Supplementary file 1 — Data S1. [file AOGS-105-805-s001.docx]

**Table S1.** Search Strategy.

1. **PubMed**

Search on February 24th, 2025

| **Search** | **Query** | **Items found** |
| --- | --- | --- |
| **#5** | #4 AND #3 | 393 |
| **#6** | #5 AND #3 | 1,025 |
| **#5** | #1 AND #2 | 3,881 |
| **#4** | placebo[MeSH Terms] OR placebo[All Fields] OR control*[All Fields] OR “no treatment”[All Fields] OR “without treatment”[All Fields] | 7,136,319 |
| **#3** | depression[MeSH Terms] OR “mood disorders”[MeSH Terms] OR mood[All Fields] OR anxiety[MeSH Terms] OR anxiety[All Fields] OR “psychological distress”[All Fields] OR “mental health”[All Fields] OR “sleep initiation and maintenance disorders”[MeSH Terms] OR insomnia[All Fields] OR “fatigue”[All Fields] OR “quality of life”[MeSH Terms] OR “quality of life”[All Fields] OR “sexual function”[All Fields] OR “sexual dysfunction”[All Fields] OR “sexual dysfunction, physiological”[MeSH Terms] OR “sexual satisfaction”[All Fields] OR “emotional changes”[All Fields] OR “adverse events”[All Fields] OR “adverse effects”[Subheading] OR “PHQ-9”[All Fields] OR “Beck Depression Inventory”[All Fields] OR “HADS”[All Fields] OR “GAD-7”[All Fields] OR “CES-D”[All Fields] OR “DASS”[All Fields] OR “SF-36”[All Fields] OR “SF-12”[All Fields] OR “EQ-5D”[All Fields] OR “WHOQOL-BREF”[All Fields] OR “EHP-30”[All Fields] OR “PROMIS”[All Fields] OR “FSFI”[All Fields] OR “IIEF”[All Fields] OR “BSFI”[All Fields] OR “FSDS”[All Fields] OR “PISQ-12”[All Fields] | 5,016,221 |
| **#2** | “contraceptives, oral, hormonal”[MeSH Terms] OR “oral contraceptives”[All Fields] OR “hormonal contraceptives”[All Fields] OR “contraceptives, oral, combined”[MeSH Terms] OR “combined oral contraceptives”[All Fields] OR “progestins”[MeSH Terms] OR “progestin*”[All Fields] OR “progestogen-only contraceptives”[All Fields] OR “progestogen-only pills”[All Fields] OR “progestin-only contraceptives”[All Fields] OR “hormonal therapy”[All Fields] OR “estrogen therapy”[All Fields] OR “estradiol”[MeSH Terms] OR “estradiol”[All Fields] OR “dienogest”[All Fields] OR “norethindrone”[MeSH Terms] OR “norethindrone”[All Fields] OR “medroxyprogesterone acetate”[MeSH Terms] OR “medroxyprogesterone”[All Fields] OR “drospirenone”[All Fields] OR “etonogestrel”[All Fields] | 200,094 |
| **#1** | endometriosis[MeSH Terms] OR endometriosis[All Fields] OR endometrioma[MeSH Terms] OR endometrioma[All Fields] OR adenomyosis[MeSH Terms] OR adenomyosis[All Fields] | 39,445 |

Updated search on August 1st, 2025

| **Search** | **Query** | **Items found** |
| --- | --- | --- |
| **#5** | #4 AND #3 | 413 |
| **#6** | #5 AND #3 | 1,064 |
| **#5** | #1 AND #2 | 3,997 |
| **#4** | placebo[MeSH Terms] OR placebo[All Fields] OR control*[All Fields] OR “no treatment”[All Fields] OR “without treatment”[All Fields] | 7,308,929 |
| **#3** | depression[MeSH Terms] OR “mood disorders”[MeSH Terms] OR mood[All Fields] OR anxiety[MeSH Terms] OR anxiety[All Fields] OR “psychological distress”[All Fields] OR “mental health”[All Fields] OR “sleep initiation and maintenance disorders”[MeSH Terms] OR insomnia[All Fields] OR “fatigue”[All Fields] OR “quality of life”[MeSH Terms] OR “quality of life”[All Fields] OR “sexual function”[All Fields] OR “sexual dysfunction”[All Fields] OR “sexual dysfunction, physiological”[MeSH Terms] OR “sexual satisfaction”[All Fields] OR “emotional changes”[All Fields] OR “adverse events”[All Fields] OR “adverse effects”[Subheading] OR “PHQ-9”[All Fields] OR “Beck Depression Inventory”[All Fields] OR “HADS”[All Fields] OR “GAD-7”[All Fields] OR “CES-D”[All Fields] OR “DASS”[All Fields] OR “SF-36”[All Fields] OR “SF-12”[All Fields] OR “EQ-5D”[All Fields] OR “WHOQOL-BREF”[All Fields] OR “EHP-30”[All Fields] OR “PROMIS”[All Fields] OR “FSFI”[All Fields] OR “IIEF”[All Fields] OR “BSFI”[All Fields] OR “FSDS”[All Fields] OR “PISQ-12”[All Fields] | 5,155,469 |
| **#2** | “contraceptives, oral, hormonal”[MeSH Terms] OR “oral contraceptives”[All Fields] OR “hormonal contraceptives”[All Fields] OR “contraceptives, oral, combined”[MeSH Terms] OR “combined oral contraceptives”[All Fields] OR “progestins”[MeSH Terms] OR “progestin*”[All Fields] OR “progestogen-only contraceptives”[All Fields] OR “progestogen-only pills”[All Fields] OR “progestin-only contraceptives”[All Fields] OR “hormonal therapy”[All Fields] OR “estrogen therapy”[All Fields] OR “estradiol”[MeSH Terms] OR “estradiol”[All Fields] OR “dienogest”[All Fields] OR “norethindrone”[MeSH Terms] OR “norethindrone”[All Fields] OR “medroxyprogesterone acetate”[MeSH Terms] OR “medroxyprogesterone”[All Fields] OR “drospirenone”[All Fields] OR “etonogestrel”[All Fields] | 202,087 |
| **#1** | endometriosis[MeSH Terms] OR endometriosis[All Fields] OR endometrioma[MeSH Terms] OR endometrioma[All Fields] OR adenomyosis[MeSH Terms] OR adenomyosis[All Fields] | 40,745 |

1. **Embase**

Search on February 24th, 2025

| **Search** | **Query** | **Items found** |
| --- | --- | --- |
| **#5** | #4 AND #3 | 701 |
| **#6** | #5 AND #3 | 2,317 |
| **#5** | #1 AND #2 | 10,578 |
| **#4** | 'placebo'/exp OR placebo:ti,ab OR control*:ti,ab OR 'no treatment':ti,ab OR 'without treatment':ti,ab | 6,899,248 |
| **#3** | 'depression'/exp OR depression:ti,ab OR 'mood disorder'/exp OR 'mood disorder':ti,ab OR mood:ti,ab OR 'anxiety'/exp OR anxiety:ti,ab OR 'psychological distress':ti,ab OR 'mental health':ti,ab OR 'sleep disorder'/exp OR insomnia:ti,ab OR fatigue:ti,ab OR 'quality of life'/exp OR 'quality of life':ti,ab OR 'sexual function':ti,ab OR 'sexual dysfunction'/exp OR 'sexual dysfunction':ti,ab OR 'sexual satisfaction':ti,ab OR 'emotional change':ti,ab OR 'adverse event':ti,ab OR 'adverse effect'/exp)) OR (('phq-9':ti,ab OR 'beck depression inventory':ti,ab OR 'hads':ti,ab OR 'gad-7':ti,ab OR 'ces-d':ti,ab OR 'dass':ti,ab OR 'sf-36':ti,ab OR 'sf-12':ti,ab OR 'eq-5d':ti,ab OR 'whoqol-bref':ti,ab OR 'ehp-30':ti,ab OR 'promis':ti,ab OR 'fsfi':ti,ab OR 'iief':ti,ab OR 'bsfi':ti,ab OR 'fsds':ti,ab OR 'pisq-12':ti,ab | 3,759,389 |
| **#2** | 'oral hormonal contraceptive'/exp OR 'oral contraceptive':ti,ab OR 'hormonal contraceptive':ti,ab OR 'combined oral contraceptive'/exp OR 'combined oral contraceptive':ti,ab OR 'progestin'/exp OR progestin*:ti,ab OR 'progestogen-only contraceptive':ti,ab OR 'progestogen-only pill':ti,ab OR 'hormonal therapy':ti,ab OR 'estrogen therapy':ti,ab OR 'estradiol'/exp OR estradiol:ti,ab OR dienogest:ti,ab OR norethindrone:ti,ab OR 'medroxyprogesterone acetate'/exp OR medroxyprogesterone:ti,ab OR drospirenone:ti,ab OR etonogestrel:ti,ab | 363,836 |
| **#1** | 'endometriosis'/exp OR endometriosis:ti,ab OR 'endometrioma'/exp OR endometrioma:ti,ab OR 'adenomyosis'/exp OR adenomyosis:ti,ab | 64,468 |

Updated search on August 1st, 2025

| **Search** | **Query** | **Items found** |
| --- | --- | --- |
| **#5** | #4 AND #3 | 801 |
| **#6** | #5 AND #3 | 2,522 |
| **#5** | #1 AND #2 | 11,182 |
| **#4** | 'placebo'/exp OR placebo:ti,ab OR control*:ti,ab OR 'no treatment':ti,ab OR 'without treatment':ti,ab | 7,287,048 |
| **#3** | 'depression'/exp OR depression:ti,ab OR 'mood disorder'/exp OR 'mood disorder':ti,ab OR mood:ti,ab OR 'anxiety'/exp OR anxiety:ti,ab OR 'psychological distress':ti,ab OR 'mental health':ti,ab OR 'sleep disorder'/exp OR insomnia:ti,ab OR fatigue:ti,ab OR 'quality of life'/exp OR 'quality of life':ti,ab OR 'sexual function':ti,ab OR 'sexual dysfunction'/exp OR 'sexual dysfunction':ti,ab OR 'sexual satisfaction':ti,ab OR 'emotional change':ti,ab OR 'adverse event':ti,ab OR 'adverse effect'/exp)) OR (('phq-9':ti,ab OR 'beck depression inventory':ti,ab OR 'hads':ti,ab OR 'gad-7':ti,ab OR 'ces-d':ti,ab OR 'dass':ti,ab OR 'sf-36':ti,ab OR 'sf-12':ti,ab OR 'eq-5d':ti,ab OR 'whoqol-bref':ti,ab OR 'ehp-30':ti,ab OR 'promis':ti,ab OR 'fsfi':ti,ab OR 'iief':ti,ab OR 'bsfi':ti,ab OR 'fsds':ti,ab OR 'pisq-12':ti,ab | 4,037,894 |
| **#2** | 'oral hormonal contraceptive'/exp OR 'oral contraceptive':ti,ab OR 'hormonal contraceptive':ti,ab OR 'combined oral contraceptive'/exp OR 'combined oral contraceptive':ti,ab OR 'progestin'/exp OR progestin*:ti,ab OR 'progestogen-only contraceptive':ti,ab OR 'progestogen-only pill':ti,ab OR 'hormonal therapy':ti,ab OR 'estrogen therapy':ti,ab OR 'estradiol'/exp OR estradiol:ti,ab OR dienogest:ti,ab OR norethindrone:ti,ab OR 'medroxyprogesterone acetate'/exp OR medroxyprogesterone:ti,ab OR drospirenone:ti,ab OR etonogestrel:ti,ab | 374,221 |
| **#1** | 'endometriosis'/exp OR endometriosis:ti,ab OR 'endometrioma'/exp OR endometrioma:ti,ab OR 'adenomyosis'/exp OR adenomyosis:ti,ab | 67,539 |

1. **Scopus**

Search on February 24th, 2025

| **Search** | **Query** | **Items found** |
| --- | --- | --- |
| **#5** | #4 AND #3 | 808 |
| **#6** | #5 AND #3 | 1,683 |
| **#5** | #1 AND #2 | 8,443 |
| **#4** | TITLE-ABS-KEY(placebo OR control* OR "no treatment" OR "without treatment") | 19,216,792 |
| **#3** | TITLE-ABS-KEY(depression OR "mood disorder" OR mood OR anxiety OR "psychological distress" OR "mental health" OR insomnia OR fatigue OR "quality of life" OR "sexual function" OR "sexual dysfunction" OR "sexual satisfaction" OR "emotional changes" OR "adverse events" OR "adverse effects" OR "phq-9" OR "beck depression inventory" OR "hads" OR "gad-7" OR "ces-d" OR "dass" OR "sf-36" OR "sf-12" OR "eq-5d" OR "whoqol-bref" OR "ehp-30" OR "promis" OR "fsfi" OR "iief" OR "bsfi" OR "fsds" OR "pisq-12") | 3,847,732 |
| **#2** | TITLE-ABS-KEY("oral hormonal contraceptive" OR "oral contraceptive" OR "hormonal contraceptive" OR "combined oral contraceptive" OR progestin* OR "progestogen-only contraceptive" OR "progestogen-only pill" OR "hormonal therapy" OR "estrogen therapy" OR estradiol OR dienogest OR norethindrone OR "medroxyprogesterone acetate" OR drospirenone OR etonogestrel) | 323,686 |
| **#1** | TITLE-ABS-KEY(endometriosis OR endometrioma OR adenomyosis) | 55,949 |

Updated search on August 1st, 2025

| **Search** | **Query** | **Items found** |
| --- | --- | --- |
| **#5** | #4 AND #3 | 835 |
| **#6** | #5 AND #3 | 1,739 |
| **#5** | #1 AND #2 | 8,614 |
| **#4** | TITLE-ABS-KEY(placebo OR control* OR "no treatment" OR "without treatment") | 19,531,841 |
| **#3** | TITLE-ABS-KEY(depression OR "mood disorder" OR mood OR anxiety OR "psychological distress" OR "mental health" OR insomnia OR fatigue OR "quality of life" OR "sexual function" OR "sexual dysfunction" OR "sexual satisfaction" OR "emotional changes" OR "adverse events" OR "adverse effects" OR "phq-9" OR "beck depression inventory" OR "hads" OR "gad-7" OR "ces-d" OR "dass" OR "sf-36" OR "sf-12" OR "eq-5d" OR "whoqol-bref" OR "ehp-30" OR "promis" OR "fsfi" OR "iief" OR "bsfi" OR "fsds" OR "pisq-12") | 3,929,114 |
| **#2** | TITLE-ABS-KEY("oral hormonal contraceptive" OR "oral contraceptive" OR "hormonal contraceptive" OR "combined oral contraceptive" OR progestin* OR "progestogen-only contraceptive" OR "progestogen-only pill" OR "hormonal therapy" OR "estrogen therapy" OR estradiol OR dienogest OR norethindrone OR "medroxyprogesterone acetate" OR drospirenone OR etonogestrel) | 326,536 |
| **#1** | TITLE-ABS-KEY(endometriosis OR endometrioma OR adenomyosis) | 56,946 |

**Figure S1.** Risk of bias assessment according to study design: a) ROBINS-I for observational studies; b) RoB2 for randomized controlled studies.

a)


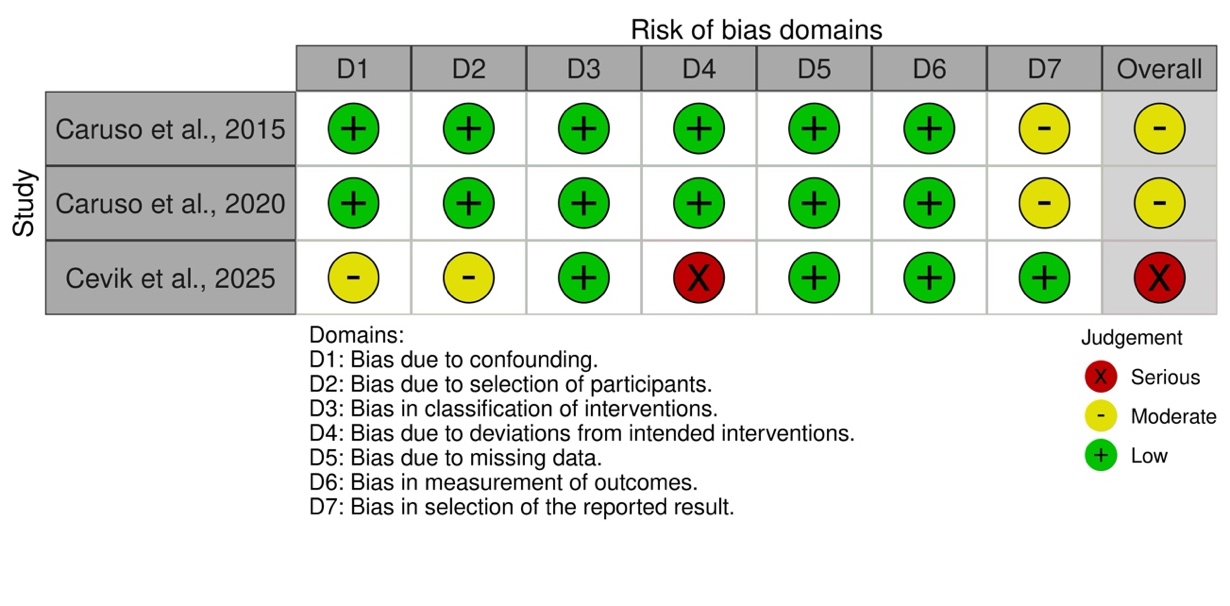


b)


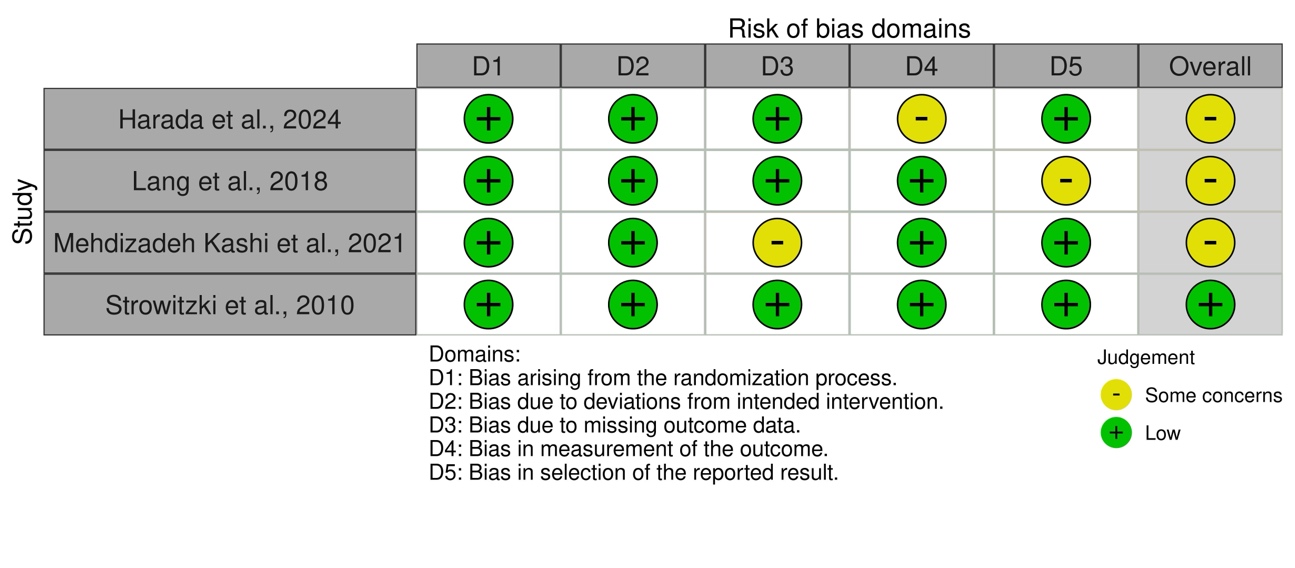


**Table S2.** ‘Absolute’ trustworthiness criteria for meta-analysis of randomized controlled studies (OGEIG, 2024)

| **‘Absolute’ RCT trustworthiness criteria (OGEIG, 2024)** | | | | | | |
| --- | --- | --- | --- | --- | --- | --- |
| **Study author, publication year** | **Governance** | | | | **Outcome** | **Does the article meet criteria?** |
|  | **Retraction^a^** | **Registration^b^** | **Ethics^c^** | **CONSORT^d^** | **Outcome variation^e^** |  |
| Harada *et al*., 2024 | No | Yes | Yes* | Not declared, but flow-diagram and checklist checked | No | Yes |
| Lang *et al*., 2018 | No | Yes | Yes | Not declared, but flow-diagram and checklist checked | No | Yes |
| Mehdizadeh Kashi *et al.,* 2022 | No | Yes | Yes | Not declared | No | Yes, with concerns |
| Strowitzki *et al.*, 2010 | No | No (but commencing prior to 2010) | Yes | No (but commencing prior to 2010) | No | Yes |

Note: COI, conflict of interest CONSORT, CONsolidated Standards of Reporting Trials; RCT, randomized controlled trial.

*Sponsored study, but declared in COI.

*^a^ “The RCT has not been retracted. (checked Pubmed and RetractionWatch*)*"*

*^b^ "The RCT was pre-registrered in a publicly available international clinical trials registry (for RCTs commencing ≥ 2010)."*

*^c^ "The RCT was approved by an ethics committee or an institutional review board (IRB).”*

*^d^ "Statement that CONSORT guidance was followed (for RCTs commencing ≥ 2010)."*

*^e^ "The primary outcome of RCT is consistent with the stated primary outcome in the RCT registration."*

**Table S3.** Summary of results a) observational studies (n=3); b) randomized-controlled studies (n=4).

a)

| **First author, year** | **Investigated treatment** | **Sample size** | | | **Outcomes** | | **Efficacy of intervention:**  **Pain-related benefit** |
| --- | --- | --- | --- | --- | --- | --- | --- |
|  |  | **Total** | **Intervention** | **Comparators** | **Psycological** | **Sexual** |  |
| Caruso *et al*., 2015 | Dienogest 2 mg/daily for 6-months (with FU at 3- and 6-months) | Baseline:  N= 92  3-month FU:  N=81  6-month FU:  N=79 | Baseline:  n= 54  3-month FU:  n= 51  6-month FU:  n= 49 | Baseline:  n= 38  3-month FU:  n= 30  6-month FU:  n= 30 | 1. 3-month FU:  - significantly lower scores in SF-36 subscale on social function (p=0.001) in intervention vs. comparator  1. 6-month FU:  - significantly lower scores in all SF-36 subscales on mental aspects in intervention vs. comparator   - mental health (p=0.001)   - emotional role (p=0.001)   - social function (p=0.001)   - vitality (p=0.001) | 1. 3-month FU:  - significantly lower scores in FSFI item on dyspareunia (p=0.002) in intervention vs. comparator  1. 6-month FU:  - significantly lower scores in all FSFI items in intervention vs. comparator   - desire (p=0.02)   - arousal   (p=0.02)   - - lubrication (p=0.04)   - orgasm (p=0.004)   - satisfation (p=0.003)   - dyspareunia (p=0.001) - significantly lower FSFI total score (p=0.001) in intervention vs. comparator - significantly lower FSDS total score (p=0.001) in intervention vs. comparator | 1. 3-month and 6-month FU:  - significant reduction in VAS for pelvic pain, dysmenorrhea, dyspareunia in treatment   Note: amenorrhea achieved at  6-month FU by n=39 (79.6%) |
| Caruso *et al*., 2020 | COCs containing 1.5 mg E2 and 2.5 mg NOMAC for 6-months | Baseline:  N= 162  3-month FU:  N=81  6-month FU:  N=79 | Baseline:  n= 99  3-month FU:  n= 89  6-month FU:  n= 77 | Baseline:  n= 63  3-month FU:  n= 57  6-month FU:  n= 39 | 1. 3-month FU:  - no differences in intervention vs. comparator  1. 6-month FU:  - significantly lower scores at total SF-36 for mental aspects in intervention vs. comparator | 1. 3-month FU:  - significantly lower FSFI total scores (p<0.001) in intervention vs. comparator - significantly lower FSDS total scores (p<0.001) in intervention vs. comparator  1. 6-month FU:  - increase in the frequency of sexual activity in intervention (p<0.001), but no between-groups differences - Significant reduction in VAS for dyspareunia in intervention vs. comparator | 1. 3-month and 6-month FU:  - significant reduction in VAS for pelvic pain, dysmenorrhea, dyspareunia in treatment; significant between-groups differences (p<0.001)   Note:  17 (17.2%) and 29 (29.3%) achieved amenorrhea at  the 3-month FU and the 6-month FU; spotting causing discontinuation in n=16 (16.2%) |
| Cevik nad Taylor*,* 2025 | Any COCs, for any duration during the retrospective study period | N= 2,682 | n= 2,006 (1,462 using COCs, 544 using  progestin-only OCs);  [n= 1,130 (56.3%) underwent surgery during the study period] | n= 676  [n= 216 (31.9%) underwent surgery during the study period] | - Depression status present: 759 (37.8%) in treatment vs. 143 (21.2%) in comparators (p<0.01) - Treatment discontinuation because of mood lability/depression: n= 300 (33.9%) | - Treatment discontinuation because of decreased libido: n= 3 (0.3%) | - Treatment discontinuation because of inadequate symptom relief: n= 463 (52.3%) |

Abbreviations: COCs, combined hormonal contraceptives; FSDS, Female Sexual Distress Scale; FSFI, Female Sexual Function Index; FU, follow-up; NOMAC, Nomegestrol Acetate; SF-36, Short Form-36 Health Survey; VAS, visual analogue scale.

b)

| **First author, year** | **Investigated treatment** | **Sample size** | | | **Outcomes** | | **Efficacy of intervention: Pain-related benefit** |
| --- | --- | --- | --- | --- | --- | --- | --- |
|  |  | **Total** | **Intervention** | **Comparators** | **Psycological** | **Sexual** |  |
| Harada *et al.*, 2024 | COCs containing 15 mg E4 and 3 mg DRSP, 24+4 regimen for 6 cycles | Randomized:  N= 162  (full-analysis set:  N= 162*)  Per-protocol:  N= 152 | Randomized:  n= 79  (full-analysis set:  n= 79*)  Per-protocol:  n= 75 | Randomized:  n= 83  (full-analysis set:  n= 83*)  Per-protocol:  n= 77 | - AE rate of psychiatric disorders (including abulia, libido decreased, insomnia): 3 (3.8%) vs. 3 (3.6%) in treatment vs. placebo (p=ns) - No discontinuation due to mood liabity/depression - No between-groups differences in dyspareunia | | - Significant reduction in all pain domains in the treatment group, with significant between-groups differences (for >=50% VAS: p<0.001) - Significant improvements in cul-de-sac induration, pelvic tender ness, and uterine mobility   Note: during withdrawal/  menstrual bleeding event and interwithdrawal/intermenstrual bleeding events occurring commonly but decreased with  treatment cycles (intermenstrual bleeding/spotting for <1 day after the second treatment cycle but persisting at study end in 24.4%) |
| Lang *et al*., 2018 | Dienogest 2 mg/daily, continuous regimen for 6 months | Randomized:  N= 262  (full-analysis set:  N= 255*)  Per-protocol:  n= 173 | Randomized:  n= 130  (full-analysis set:  n= 126*)  Per-protocol:  n= 85 | Randomized:  n= 132  (full-analysis set:  n= 129*)  Per-protocol:  n= 88 | - SF-36 mental health component score: improvement in treatment 1.86 vs. worsening in placebo (- 0.87) - No discontinuation due to mood liabity/depression | - Significant reduction in B&B scores for dyspareunia in treatment vs. placebo | - Significant reduction in pelvic pain and B&B scores, with significant between-groups differences (p<0.0001) - Decreased intake of pain-killers in treatment vs. increase in placebo   Note: amenorrhea achieved at  6-month FU by 17.6%; heavy bleeding in 7.4% |
| Mehdizadeh Kashi et *al.,* 2022 | Dienogest 2 mg/daily, continuous regimen for 6 months | Randomized:  N= 108  Per-protocol:  N= 89 | Randomized:  n= 36  Per-protocol:  n= 30 | Randomized:  n= 36  Per-protocol:  n= 29 | - Psycological health subscale: improvement in dienogest arm by 5.67±1.23; in COCs arm by 4.95±3.08; in placebo arm by 1.05±0.43 (p=0.002) - No discontinuation due to mood liabity/depression | - Significant reduction in VAS for dyspareunia in dienogest vs. placebo (p=0.03), or vs. COCs (p=0.001); no between-treatment arms differences | - VAS for pelvic pain significantly higher in placebo vs. dienogest (p= 0.04), or vs. COCs (p< 0.001); no between-treatment arms differences   Note: Spotting/bleeding in 20% treated by dienogest and 13.3% treated by COCs |
|  | COCs containing 0.03 mg EE and 0.3 mg LNG, mg/daily, continuous regimen for 6 months |  | Randomized:  n= 36  Per-protocol:  n= 30* |  |  |  |  |
| Strowitzki *et al.,* 2010 | Dienogest 2 mg/daily, continuous regimen for 3 months, after a 4-weeks treatment-free period | Randomized:  N= 198  (full-analysis set:  N= 198*)  Per-protocol:  N= 144 | Randomized:  n= 102  (full-analysis set:  n= 102*)  Per-protocol: n= 74 | Randomized:  n= 96  (full-analysis set:  n= 96*)  Per-protocol: n= 70 | - SF-36 mental health component score: no difference; - SF-36 role emotional domain: improvement in treatment vs. placebo (18.4 +/-33.9%, treatment; 9.6 +/- 46.4%, placebo) - No discontinuation due to mood liabity/depression - AE rate of depression: 2 (2%) vs. 2 (2.1%) in treatment vs. placebo (p=ns) | - Significant reduction in B&B scores for dyspareunia in treatment vs. placebo | - Significant reduction in pelvic pain and B&B scores, with significant between-groups differences (p<0.0001)   Note: amenorrhea achieved at  3-month FU by 1%; proportions of women with ‘‘infrequent bleeding’’ (18.4%), ‘‘frequent bleeding’’ (19.4%), ‘‘irregular bleeding’’ (37.8%), ‘‘prolonged bleeding’’ (24.5%) and ‘‘normal bleeding’’ (23.5%); no drop-outs by bleeding patterns |

Abbreviations: B&B, Biberoglu and Behrman scale; COCs, combined hormonal contraceptives; DRSP, Drospirenone; E4, Estetrol; EE, Ethinylestradiol; FSDS, Female Sexual Distress Scale; FSFI, Female Sexual Function Index; FU, follow-up; LNG, Levonorgestrel; NOMAC, Nomegestrol Acetate; SF-36, Short Form-36 Health Survey; VAS, visual analogue scale.

* Reported results based on full-analysis set.
